# Effects of Dandelion Extracts on the Ruminal Microbiota, Metabolome, and Systemic Inflammation in Dairy Goats Fed a High-Concentrate Diet

**DOI:** 10.3390/vetsci13010028

**Published:** 2025-12-27

**Authors:** Mussa Suleiman Mgeni, Li Zhang, Yu Chen, Xianwen Dong, Ziqing Xiu, Junqiu Zhang, Juncai Chen, Yawang Sun

**Affiliations:** 1 College of Animal Science and Technology, Southwest University, Chongqing 400716, China; 2 Department of Animal Science and Aquaculture, School of Agriculture, State University of Zanzibar (SUZA), Zanzibar 71210, Tanzania; 3 Chongqing Academy of Animal Sciences, Chongqing 402460, China

**Keywords:** high-concentrate diet, dairy goats, dandelion extracts, microbiome, metabolome, inflammation

## Abstract

This study examined the effect of dandelion extracts on the ruminal microbiota, metabolome, and inflammatory response in dairy goats fed a high-concentrate diet. Eighteen Guanzhong dairy goats were assigned to three groups: low-concentrate diet group (L group, F:C = 60:40), high-concentrate diet group (H group, F:C = 35:65), and dandelion group (D) with 1% dandelion extracts based on the H group. Over six weeks, milk, blood, and ruminal fluid were collected to analyze pH, volatile fatty acids (VFAs), inflammatory markers, microbiome, and metabolome. Feed intake was significantly higher in the H and D groups than in the L group. Ruminal pH decreased in the H group, acetate was significantly higher in the L and D groups, and serum IgA was higher in D group. *Bacteroidetes* and *Firmicutes* were dominant rumen phyla, and dandelion supplementation significantly increased *Proteobacteria*. *Prevotella* abundance was enriched in the L and D groups and showed a positive correlation with acetate. Dandelion extracts also increased the production of trans-3-Hydroxy-L-proline and 7-Ethyl-5,6-dihydro-1,4-dimethylazulene in the rumen while improving amino acid and lipid metabolism. Overall, supplementation of dandelion extracts positively influenced rumen microbiota, enhanced production of beneficial metabolites, and positively influenced immune function in dairy goats fed high-concentrate diet.

## Simple Summary

This study showed that the addition of dandelion extracts to a high-concentrate diet for dairy goats offers significant benefits by increasing ruminal pH and improving fermentation by enhancing the levels of acetate in the rumen. Additionally, dandelion extracts bolstered the immune response through increased serum IgA. Also, dandelion extracts lowered inflammatory markers in the mammary gland and increased the growth of good ruminal microflora, especially *Prevotella*. This increased the production of good ruminal metabolites and made the metabolism of amino acids and fats better. Overall, these findings underscore the role of dandelion extracts used as a feed additive in supporting rumen health and promoting beneficial microbial and metabolic profiles in dairy goats under a high-concentrate diet.

## 1. Introduction

In intensive dairy production systems, while milk yield remains the primary economic determinant, nutritional management of ruminants constitutes a critical factor influencing both production efficiency and animal health status. The widespread implementation of high-concentrate diets aims to address the elevated energy demands of lactating ruminants [1] but concurrently elevates risks of metabolic dysregulation. Accelerated fermentation of non-structural carbohydrates in these diets promotes rapid accumulation of organic acid and short-chain fatty acids, which make ruminants more likely to get subacute ruminal acidosis [2]. Once acidosis occurs, lipopolysaccharides (LPS) are liberated into the rumen from the outer membranes of lysed Gram-negative bacteria and then migrate into the circulatory system through compromised ruminal epithelial barrier [3]. After penetrating the mammary tissue, LPS generates inflammatory reactions that reduce the milk yield [4]. Beyond binding to Toll-like receptor 4 and initiating innate immune responses [5], LPS also stimulate the release of various inflammatory mediators, contributes to systemic inflammation, and disrupts metabolic and immune homeostasis in ruminants [3]. Lactating dairy goats are particularly susceptible to high-concentrate diets due to their rapid ruminal fermentation rates and limited buffering capacity, which predispose them to subacute ruminal acidosis and systemic inflammation.

Livestock producers have employed several dietary strategies, including antibiotics, to counteract metabolic disturbances induced by high-concentrate feeding regimes [6]. Nevertheless, the global emergence of antimicrobial resistance has prompted stringent regulatory prohibitions on antibiotics in livestock production [7]. This paradigm shift has accelerated research into phytogenic feed additives, particularly plant-derived bioactive compounds, as sustainable alternatives for enhancing production efficiency and animal welfare [8]. Studies have demonstrated that plant extracts contain a variety of biologically effective chemicals, such as flavonoids, that possess antibacterial, antioxidant, and anti-inflammatory properties [9]. These chemicals can improve productive efficiency and reduce inflammation in ruminants feeding high-concentrate diets [10].

Dandelion (*Taraxacum officinale*), a perennial herb belonging to the Asteraceae family, is widely distributed in temperate regions of the Northern Hemisphere. This species contains a variety of phytochemicals, such as flavonoids, which have been reported to have diverse biological effects, including anti-inflammatory, antibacterial, and anti-oxidative properties [11]. Recently, several studies have investigated the potential roles of dandelion extracts in helping ruminants overcome various stresses caused by a diet high in grains. A study by Zhang et al. [12] demonstrated that dandelion extracts improved the rumen homeostasis and production of dairy cows fed a high-concentrate diet. Furthermore, dandelion extracts reduced serum levels of TNF-α and IL-8 in dairy cows, suppressing the unfavorable immunological reaction [13]. Li et al. [14] also found that dandelion extracts might improve rumen fermentation and feed utilization by raising the number of rumen *Firmicutes* bacteria and *Firmicutes*-to-*Bacteroidetes* ratio. These findings collectively highlight the potential of dandelion extracts as functional feed additives to optimize rumen function and improve dairy productivity.

Despite the above promising results in dairy cows, the effects of dandelion extracts on dairy goats remain largely unexplored, particularly regarding ruminal microbiome, metabolome, and inflammatory responses. Evaluating the ruminal microbiota and metabolome provides a holistic understanding of dietary impacts, as shifts in microbial communities directly influence fermentation patterns and metabolite profiles, which in turn modulate inflammatory responses. Therefore, this study investigated the effects of dandelion extracts on ruminal microbiome, metabolome, and inflammatory response in dairy goats fed a high-concentration diet, with the hypothesis that dandelion extracts enhance ruminal microbial diversity, modulate microbial community composition, promote beneficial metabolite production, and reduce inflammation.

## 2. Materials and Methods

### 2.1. Ethics Statement

The experiment received ethical approval from Southwest University of China’s Animal Ethics Committee for Animal Welfare (Number: 3167130267). The feeding and care of the animals were carried out in compliance with the applicable regulations.

### 2.2. Animal and Design of Experiments

The experiment was conducted at the Goat farm of Southwest University from March to May, with the average temperature ranging from 15 °C to 25 °C. The experiment involved 18 multiparous Guanzhong dairy goats with an average weight of 60.55 ± 4.25 kg, possessing comparable health conditions and a lactation duration of 90 ± 10 days. The goats were randomly allocated to 3 dietary groups with 6 replicates per group through a complete randomized design and were housed in well-ventilated barns with natural lighting. The groups were the low-concentrate diet group (L group), which served as a control group and contained a forage-concentrate ratio (F:C) of 60:40, the high-concentrate diet group (H group) had an F:C ratio of 35:65, and the dandelion group (D group), which contained 1% dandelion extracts based on H group, following the dose applied by Zhang et al. [12]. The nutritional program was based on total mixed rations formulated to meet the nutrient requirements of lactating dairy goats [15]. The feeding experiment lasted for 6 weeks, with the first week serving as an adaptation period. All goats were milked twice daily at 9:00 a.m. and 5:00 p.m., and the individual milk production was documented on the final day of each week. Goats were fed twice daily at 10:00 a.m. and 6:00 p.m. with free access to water. A fixed daily allowance of TMR was offered based on body weight requirements rather than ad libitum feeding, and feed intake was monitored daily by measuring refusals from both the morning and evening feedings. Feed refusals (orts) were checked at each feeding and maintained below 5% of the offered TMR, and ort samples were not analyzed because the consistently low and uniform refusal amounts did not permit reliable compositional assessment. Approximately 1 kg of feed from each dietary group was collected, sealed in sterile nylon bags, and stored at −20 °C for subsequent analysis using the method described in [12]. The ingredient composition and nutrient profile of the three diets are presented in Table 1.

### 2.3. Sample Collection and Assessment

During the experiment, 50 mL of goat milk samples was collected on the last day of each week for analysis of milk protein, fat, solids, and lactose, which were determined using a lactoscan analyzer, and a 500 mL aliquot of milk from the last week was collected and stored at −20 °C for RNA extraction. Blood from goats was collected from jugular vein for serum, centrifuged at 4 °C and 3500 rpm for 10 min, and stored at −80 °C for measurement of immune and pro-inflammatory indicators. Goats’ rumen fluid was collected two hours after morning feeding using a sterile oral stomach tube attached to sterile syringe. The first 20 mL was discarded to reduce salivary contamination, and 100 mL was collected and divided into two portions for microbiota (strained through 4 layers of sterile gauze) and pH analysis. For metabolome analysis, the strained fluid was centrifuged at 6000× *g* for 15 min, filtered through a 0.22-micron filter membrane, and then kept at −80 °C. Fatty acids (VFAs) and rumen LPS assays were performed using the supernatant after the strained fluid was centrifuged for 5 min at 12,000× *g*.

### 2.4. Assessment of Flavonoid Contents from Dandelion Extracts

The dandelion extracts were obtained from Daosifu Biotechnology Co., Ltd., in Nanjing, Jiangsu, China. Over 200 flavonoid metabolites in the dandelion extracts were subjected to an absolute quantitative analysis based on the targeted metabolome approach through liquid chromatography-mass spectrometry (LC-MS). Li et al.’s protocols [14] were utilized to evaluate the dandelion extracts, and Table 2 lists the concentrations of the top 20 target flavonoids.

### 2.5. Assessment of Ruminal Fluid VFA and LPS

The 1 mL sample was thawed and mixed with 200 μL of 25% (*w*/*v*) metaphosphoric acid solution and 60 μL of 21 g/L crotonic acid solution. The mixture was then incubated at 4 °C for 30 min and centrifuged at 12,000 rpm for 10 min. Immediately after centrifugation, 0.7 mL of the supernatant was then mixed with 0.7 mL of chromatographic methanol (1:1 dilution) and centrifuged at 10,000 rpm for 5 min. The supernatant was then filtered using a 0.22 μm filter membrane into a 1.5 mL EP tube for later use. Gas chromatography was used to measure the amounts of volatile fatty acids (SPL-2010plus, Shimadzu Enterprise Management (China) Co., Ltd, Shanghai, China), using a Varian GC CP3800 gas chromatograph fitted with an HP-FFAP capillary column (30 m × 0.53 mm × 1.0 μm; Agilent Technologies, Inc, Santa Clara, CA, USA). The following parameters were used to create standard curves for acetic acid, propionic acid, butyric acid, and valeric acid before sample analysis. A temperature of 250 °C was established for the injector and maintained for the detector. After starting the column temperature at 60 °C and holding it there for two minutes, it was raised linearly to 140 °C at a rate of 10 °C per minute and held there for two more minutes. The temperature was then raised to 230 °C at a rate of 20 °C per minute, with a final hold of 5 min. The carrier gas was high-quality nitrogen gas with a split ratio of 30 and a flow rate of 40 mL/min.

Kinetic Chromogenic Assay Bioendo™ KC Endotoxin Test Kit (Xiamen Bioendo Technology Co., Ltd., Xiamen, China) was used to identify the lipopolysaccharide in ruminal fluid. The obtained rumen fluid samples were centrifuged at 10,000 rpm for 5 min at 4 °C after being thawed at room temperature and a 1.5 mL sterile centrifuge tube was then filled with the supernatant. The LPS content was then determined using the processed rumen fluid sample under the kit’s instructions. A microtiter plate reader (Synegy H1, Gene Company Limited, Beijing, China) was used for assessing the optical density (OD) value at 405 nm. To determine the absorbance values of the sample holes, the standard curve formula was derived and entered into the formula.

### 2.6. Assessments of Serum Immune Indicators and Pro-Inflammatory Cytokines

The content of immunoglobulin (IgA and IgG) and pro-inflammatory cytokine (TNF-α, IL-1, IL-6, and IL-8) in serum was evaluated under the Goat ELISA Kit (JIANGSU MEIBIAO BIOLOGICAL TECHNOLOGY Co., Ltd. Nanjing, China). A microtiter plate reader (Synegy H1, Gene Company Limited, Beijing, China) was used for assessing the optical density (OD) value at 450 nm. To determine the absorbance values of the sample holes, the standard curve was generated by plotting the average O.D. (450 nm) obtained for each of the six standard concentrations on the vertical (Y) axis versus the corresponding concentration on the horizontal (X) axis and the standard curve formula was derived and entered into the formula.

### 2.7. RNA Extraction and Assessment of Gene Expression for Inflammatory Indicators

The methodology presented in the previous study [12] was used with the following equipment to extract the RNA content within goat milk mammary epithelial cells. A 50 mL centrifuge tube was filled with a milk sample, which was centrifuged for 10 min at 4 °C and 3500 rpm. The cell pellet was treated with 1x phosphate-buffered saline (Biosharp, Life Science, Beijing, China) after the top layer of fat was scraped off with a spoon, and the supernatant was also removed. The cell pellet was then washed two times and centrifuged using the same procedure. The cell pellets were collected into a 1.5 mL RNAase-free container after the last centrifugation, and RNA was extracted according to the directions of the TriQuick Total RNA Extraction Reagent (from Beijing Solarbio Science & Technology Co., Ltd., Beijing, China). Based on the A260/A280 ratio, RNA purity was assessed using NanoDrop One (Thermo Fisher Scientific, 5225 Verona Road, Madison, WI 53711, assembled in the USA). For cDNA tests and RT-PCR, only samples with an A260/A280 ratio between 1.8 and 2.1 were used.

The HiScript III RT SuperMix for qPCR (+gDNA wiper) reverse transcription kit from Vazyme Biotech Co., Ltd. (Nanjing, China) was used to reverse-transcribe mRNA to cDNA. Through the ChamQ Universal SYBR qPCR Master Mix kit (Vazyme Biotech Co., Ltd., Nanjing, China), real-time quantitative PCR was carried out in the manner described below: 30 s of initial denaturation at 95 °C, 40 cycles of the reaction for 3–10 s at 95 °C, and 10–30 s at 60 °C are also included. Melting curve at 95 °C for 15 s, 60 °C for 60 s, and 95 °C for 15 s. The relative mRNA expression levels were then measured using the 2^−ΔΔCT^ method, and the melting curves were analyzed to ensure selective amplification [12]. Chinese company Beijing Liuhe Huada Gene Technology Co., Ltd. (BGI·Write, Beijing, China) created the primers for the inflammatory genes mentioned in Table 3.

### 2.8. S Ribosomal RNA Gene Sequencing

The TGuide S96 Magnetic Soil/Stool DNA Kit (Tiangen Biotech Co., Ltd., Beijing, China) was used to extract total genomic DNA from ruminal fluid samples under the manufacturer’s instructions. The NanoDrop 2000 UV-Vis spectrophotometer (Thermo Scientific, Wilmington, MA, USA) was used to measure the concentration and purity of the extracted DNA, and electrophoresis on a 1.8% agarose gel was used to assess the quality and amount of the DNA. For the bacterial 16S rRNA gene, primer pairs 338F: 5′-ACTCCTACGGGAGGCAGCA-3′ and 806R: 5′-GGACTACHVGGGTWTCTAAT-3′ were used to amplify the hypervariable region V3–V4. To enable deep sequencing, sample-specific Illumina index sequences were used to tail the forward and reverse 16S primers. In a total reaction volume of 10 μL, the PCR was carried out using the following components: DNA template 5–50 ng, forward primer (10 μM) 0.3 μL, reverse primer (10 μM) 0.3 μL, KOD FX Neo Buffer 5 μL, and dNTP (2 mM each) 2 μL, KOD FX Neo 0.2 μL, and then ddH2O up to 20 μL. Following a 5-minute initial denaturation at 95 °C, there were 20 cycles of denaturation at 95 °C for 30 s, annealing at 50 °C for 30 s, extension at 72 °C for 40 s, and a last step at 72 °C for 7 min. The Qsep-400 (BiOptic, Inc., New Taipei City, Taiwan, ROC) was used to quantify the amplified products after they had been purified using the DNA purification kit (Omega Inc., Norcross, GA, USA). Using an Illumina Novaseq 6000 (Beijing Biomarker Technologies Co., Ltd., Beijing, China), the amplicon library was paired-end sequenced (2 × 250). One operational taxonomic unit (OTU) was assigned to the qualifying sequences that met 97% similarity standards using USEARCH (version 10.0). With a 70% confidence level, taxonomy annotation of the OTUs/ASVs was carried out using the SILVA database (version 138.1) and the Naive Bayes classifier in QIIME2. Alpha was used with QIIME2 software to determine each sample’s species diversity complexity. The diversity in samples for species complexity was evaluated using principal coordinate analysis (PCoA) to analyze beta diversity projections. The diversity and abundance of bacteria were compared using a one-way analysis of variance. An evaluation of the differentially abundant taxa was conducted using linear discriminant analysis (LDA) alongside effect size (LEfSe). The sequencing information was analyzed using the web-based tool BMKCloud (https://www.biocloud.net, accessed on 8 October 2024).

### 2.9. Assessment of Metabolome

Approximately 100 μL of each ruminal fluid sample was weighed individually, and 500 μL of extraction solvent containing an internal standard (methanol–acetonitrile volume ratio = 1:1, internal standard concentration 20 mg/L) was added. The mixture was vortexed for 30 s and then ultrasonically sonicated for 10 min in an ice water bath. The sample was then allowed to stand at −20 °C for an hour. After being centrifuged for 15 min at 4 °C and 12,000 rpm, 500 μL of the supernatant was carefully collected and placed in an EP tube. The extract was then dried in a vacuum concentrator. Immediately after the addition of 160 μL of extract (acetonitrile-water volume ratio: 1:1) to the dried metabolites for re-dissolution, the sample was vortexed for 30 s and ultrasonicated for 10 min in an ice water bath. The sample was then centrifuged at 4 °C and 12,000 rpm for 15 min, and approximately 120 μL of the supernatant was carefully removed and transferred into a 2 mL injection bottle. To monitor instrument performance, 10 μL of each sample was then mixed into QC samples, which were analyzed alongside experimental samples.

The Waters Xevo G2-XS QTof high-resolution mass spectrometer and the Waters Acquity I-Class PLUS ultra-high-performance liquid tandem make up the LC/MS system for metabolomics study. The column (1.8 µm, 2.1*100 mm) was acquired from Waters Acquity UPLC HSS T3. Under the guidance of the acquisition software (MassLynx V4.2, Waters), the Waters Xevo G2-XS QTOF high-resolution mass spectrometer is capable of gathering primary and secondary mass spectrometry data in MSe mode. Dual-channel data acquisition was able to be carried out simultaneously with low collision energy and high collision energy in each data acquisition cycle. For a mass spectrum, the scanning frequency is 0.2 s, the high collision energy range is 10–40 V, and the low collision energy remains off. The following are the ESI ion source’s parameters: cone voltage: 30 V; ion source temperature: 100 °C; desolvent gas temperature: 500 °C; backflush gas flow rate: 50 L/h; desolventizing gas flow rate: 800 L/h; capillary voltage: 2500 V (positive ion mode) or −2000 V (negative ion mode). Based on the online METLIN database and Biomark’s self-built library for identification, Progenesis QI software processes the raw data obtained using MassLynx V4.2 for peak extraction, peak alignment, and other data processing tasks.

### 2.10. Statistical Analysis

The study used IBM SPSS Statistics version 23 and Graph Pad Prism version 9.0.0 to analyze parameters and diagrams. Variability between treatments was assessed using one-way ANOVA and Duncan’s multiple comparisons. Microbiome diversity and bacteria abundance were compared using one-way ANOVA. Prior to ANOVA, normality of data distribution was assessed using the Shapiro–Wilk test, and homogeneity of variances was verified with Levene’s test. Linear discriminant analysis and effect size were used to evaluate differentially abundant taxa. Sequencing information was analyzed using BMKCloud. Metabolome repeatability was evaluated using principal component analysis and Spearman correlation analysis. Databases like KEGG, HMDB, and Lipidmaps were examined for classification and pathway information. OPLS-DA modeling was performed using R and 200 permutation tests. Correlations were determined using Spearman’s correlation computation method and Pearson’s. Statistical significance was defined as *p* < 0.05.

## 3. Results

### 3.1. Influence of Various Feeds on Feed Intake, Milk Yield, and Milk Quality in Dairy Goats

**Figure 1:**
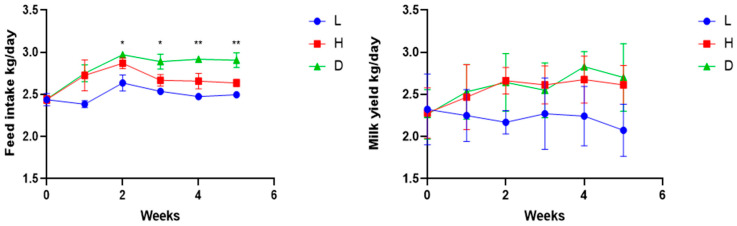
Influence of various feeds on feed intake (**left panel**) and milk yield (**right panel**) in dairy goats. The abbreviation L represents the low-concentrate diet group (control group) with an F:C of 60:40; H represents the high-concentrate diet group with an F:C of 35:65; and D represents the dandelion group with a % dandelion extracts group based on the H group; * shows a significant (*p* < 0.05) difference between the groups; ** shows a highly significant (*p* < 0.01) difference between the groups.

From weeks 2 to 3, the feed intake of the L group was significantly lower (*p* < 0.05) compared to the H and D groups (Figure 1). In the last 2 weeks of the experiment, feed intake of the L group remained consistently lower than that of H and D groups (*p* < 0.01). On the other hand, a notable difference in feed intake between the H and D groups was observed in the last 3 weeks of the experiment. Furthermore, no significant differences in milk production or milk quality were observed among the groups throughout the study (Figure 1 and Table 4).

### 3.2. Influence of Various Feeds on Ruminal pH, Ruminal LPS Concentrations, and Ruminal Fermentation Parameters in Dairy Goats

**Figure 2:**
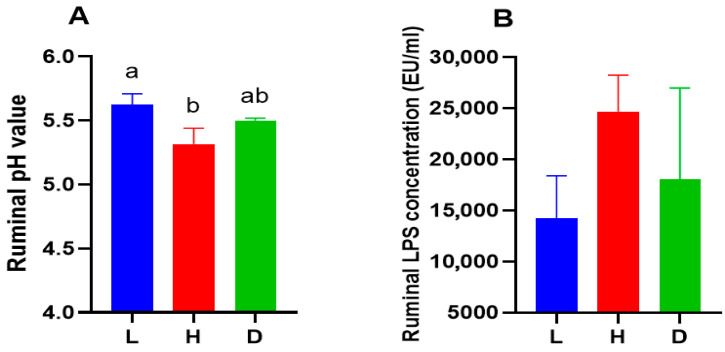
Influence of various feeds on ruminal pH (**A**) and ruminal LPS concentration (**B**) in dairy goats. The data are expressed as the mean with SEM; Various letters in a particular row of data show a significant difference (*p* < 0.05). In contrast, the presence or absence of the same letter in the superscript shows no significant difference (*p* > 0.05).

The goats in the L group exhibited a significantly (*p* < 0.05) higher ruminal pH as compared to the H group (Figure 2A), while no significant difference in ruminal LPS concentration among the groups (Figure 2B). Propionate and total VFAs were much higher for the goats in the H and D groups as compared to the L group (*p* < 0.001), and the valerate was significantly lower (*p* < 0.05) in the L group compared with the H group. Additionally, acetate levels were significantly lower in H group compared with L and D groups (*p* < 0.05), and no significance differences in butyrate were observed among the groups (Table 5).

### 3.3. Influence of Various Feeds on Serum Immunoglobulin, Serum Pro-Inflammatory Cytokines, and the Expression of a Gene Linked to Inflammation in Dairy Goats

**Figure 3:**
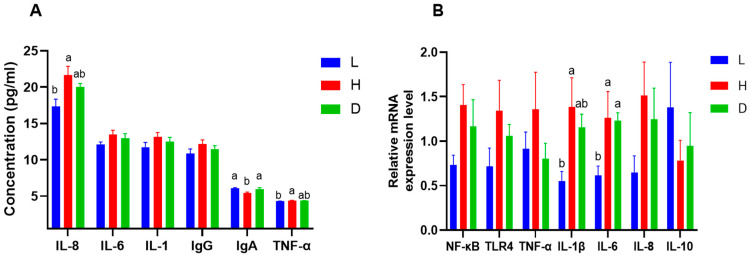
Influence of various feeds on serum immunoglobulin, serum pro-inflammatory cytokines, and the expression of a gene linked to inflammation in dairy goats. (**A**) Serum immunoglobulin and pro-inflammatory cytokines; (**B**) Gene expression linked to inflammation in milk. The data are expressed as the mean with SEM; Various letters in a particular row of data show a significant difference (*p* < 0.05). In contrast, the presence or absence of the same letter in the superscript shows no significant difference (*p* > 0.05).

The goats in the L and D groups had significantly higher levels of serum IgA than the H group (*p* < 0.05). Serum TNF-α and IL-8 levels were significantly lower in the L group compared with the H group (*p* < 0.05), while no significant differences for serum IgG, IL-1, and IL-6 (Figure 3A). Figure 3B indicated that the H group significantly increased relative mRNA expression levels of IL-1β and IL-6 (*p* < 0.05), while no significant differences in the other immune-related genes were observed between the groups.

### 3.4. Effect of Diets on Ruminal Microbial Composition in Dairy Goat

**Figure 4:**
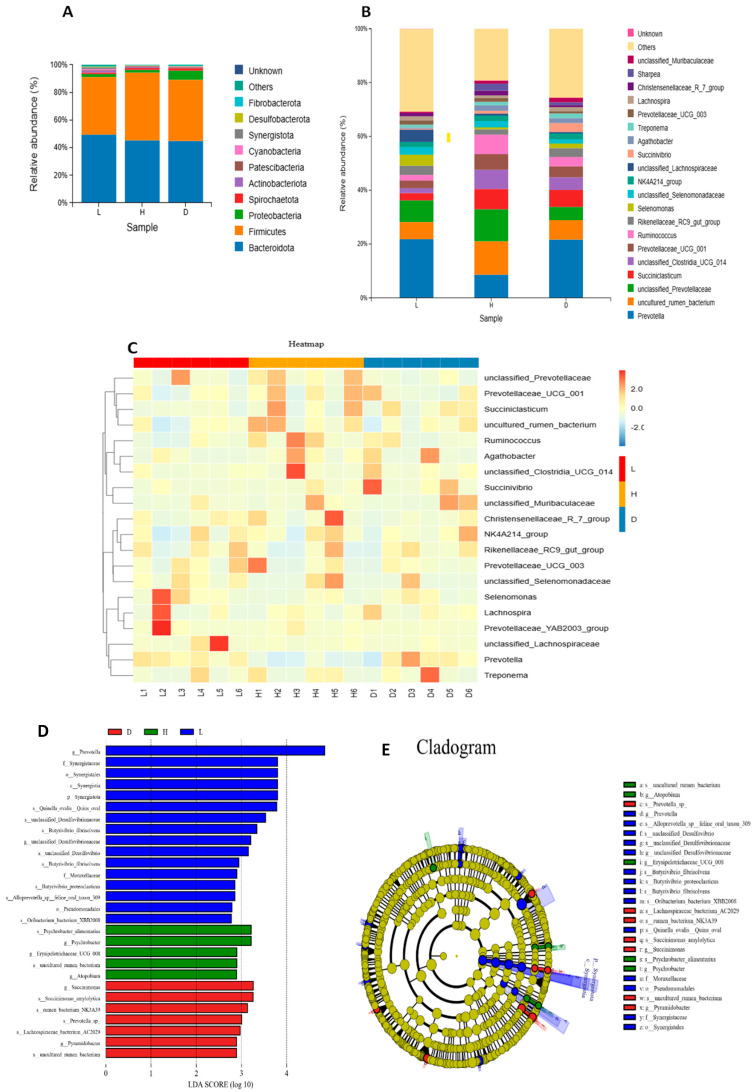
Microbial composition in dairy goats. (**A**) Relative abundance of ruminal bacteria at the phylum level; (**B**) Relative abundance of ruminal bacteria at the genus level; (**C**) Species composition heat map of dairy goat illustrating the top 20 bacteria at the genus level; the blue-to-red color gradient indicates the species’ relative abundance, which ranges from low to high. (**D**) Line Discriminant Analysis (LDA) score distribution histogram: a more significant difference is indicated by a longer bar (LDA score > 2), and the bars were colored based on which group had the largest abundance of the related feature; (**E**) Lefse analysis cladogram diagram: circles represent taxonomic levels, nodes indicate terms on the corresponding taxonomic level, and the size of the dots indicates relative abundance. Yellow indicates species with no significant difference, while nodes were colored according to the group with the highest relative abundance.

The relative abundances of the top 10 and top 20 rumen bacterial phyla and genera, respectively, were reported in Figure 4A,B. *Bacteroidota* and Firmicutes were the dominant phyla in all dietary groups. The relative abundance of *Proteobacteria* was significantly higher in the D group compared with other groups (*p* < 0.05) (Table 6). Furthermore, we identified the rumen bacterial genera with relative abundance greater than 1% as dominant; therefore, *Prevotella*, *uncultured_rumen_bacterium*, *unclassified_Prevotellaceae*, *Prevotellaceae_UCG_001*, *Succiniclasticum*, *unclassified_Clostridia_UCG_014*, and *Ruminococcus* were the dominant genera across all dietary groups. Nevertheless, the relative abundance of *Ruminococcus* was higher in H and D groups compared with L group and the addition of 1% dandelion extracts in a high-concentrate diet significantly increased the relative abundance of *Prevotella* (*p* < 0.05).

A heat map was used for species composition analysis to better analyze the variations in species composition between groups and to show the distribution trends of species abundance within each group. This technique makes it easier to identify and compare species groups across several categories, as shown in Figure 4C. The analysis reveals that genera like *Prevotella*, *Prevotellaceae_UCG_003*, and *Selenomonas* are more abundant in the L group, as indicated by red shades, and also genera like *Ruminococcus* and *NK4A214_group* are more abundant in the H group. On the other hand the addition of 1% dandelion extracts in a high-concentrate diet exhibited sole improvement in genera such as *Prevotella*, *Succiniclasticum*, and *Succinivibrio*.

Three distinct dietary categories were shown to have varying abundances of microbial species in Figure 4D,E. Numerous taxa, mostly represented by blue bars, are present in the L group. *g_Prevotella*, *f_Synergistaceae*, *o_Synergistales*, *c_Synergistia*, *p_Synergistota*, and *s_Quinella_ovalis_Quins_oval* are some of the important taxa with the highest LDA scores and a strong link to this diet. The H group reported a slight increase in microbial taxa such as *s_Psychrobacter_alimentarius*, *g_Psychrobacter*, *g_Atopobium*, and *g_Erysipelotrichaceae_UCG_008*. Furthermore, when 1% dandelion extracts were added to a high-concentration diet, it significantly stimulated taxa such as *g_Succiniomonas*, *s_Succiniomonas_amylolytica*, *s_Prevotella_sp*, *s_rumen_bacterium_NK3A39*, *s_lachnospiraeae_bacterium_AC2029*, and *g_Pyramidobacter*.

### 3.5. Influence of Various Feeds on Alpha Diversity and Beta Diversity of the Microbial Community in Dairy Goats

**Figure 5:**
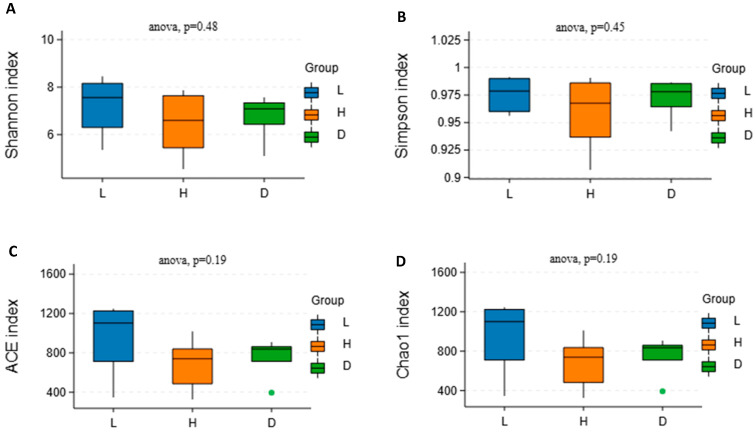

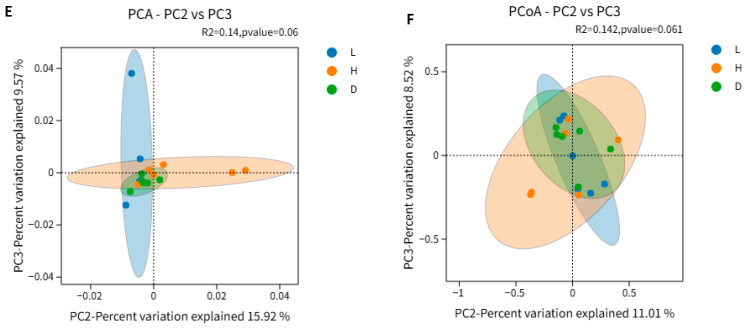
Influence of various feeds on alpha diversity indices and beta diversity analysis in dairy goats. (**A**) Shannon index; (**B**) Simpson index; (**C**) ACE index; (**D**) Chao 1 index; (**E**) PCA, Principal component analysis; (**F**) PCoA, Principal coordinates analysis. The region that contains 95% of all samples that can be inferred from the underlying Gaussian distribution is defined by the confidence ellipse.

The alpha diversity indices were higher in the L group compared with the H and D groups (Figure 5A–D); however, the difference did not reach significance. In contrast to the H group, the D group displayed a slightly higher degree of alpha diversity indices with no significance. Microbial beta diversity analysis using the Bray–Curtis algorithm was performed via PCA and PCoA analysis (Figure 5E,F), where it was observed that the confidence ellipses for all groups displayed substantial intersection; nevertheless, a broader spread in PCoA compared with the PCA graph suggested minimal variation in dairy goat’s rumen microbial diversity among the groups.

### 3.6. Influence of Various Feeds on Dairy Goat Rumen Fluid Metabolites

**Figure 6:**
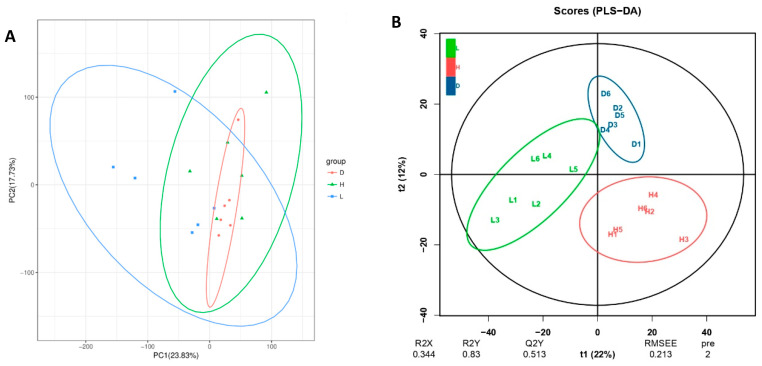

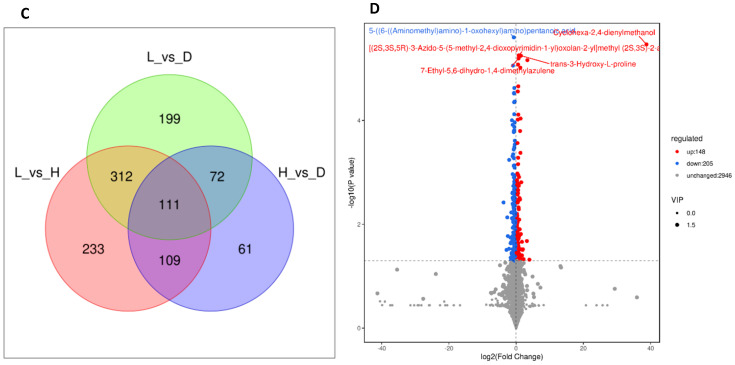
Rumen fluid metabolome analysis for the dairy goats fed three different diets. (**A**) Principal component analysis (PCA): Samples from the same group are represented by the same color, while samples from other groups are labeled with various colors, and each point in the plot represents a sample. (**B**) Partial least squares discriminant analysis (PLS-DA): Below the graph are annotations for the model parameters, which include R2X, R2Y, Q2Y, RMSEE (root mean square error of estimate), pre (number of predictive components), and ort (number of orthogonal components). (**C**) Venn diagram: A comparison group is symbolized by each circle in the figure; the numbers outside the overlapping areas show the number of distinct differential metabolites for each comparison group, while the numbers in the overlapping areas between circles show the number of differential metabolites shared by the comparison groups. (**D**) Volcano plot: The Variable Importance in Projection, or VIP, is represented by the size of each data point in the volcano plot, with blue points representing down-regulated metabolites, red points representing up-regulated metabolites, and gray points representing detected metabolites without significant differences.

The LC-QTOF technology served as the foundation for the present study, which included the qualitative and quantitative metabolomics assessment of 18 samples. The metabolites found using the standard approach are shown in this study result. A total of 17,165 peaks were found, and 3299 metabolites were identified. Organic acids, amino acids, fats, amines, and carbohydrates were among these metabolite types. Based on the PCA analysis (Figure 6A), the L group exhibits the greatest dispersion, as shown by the blue ellipse, and can be clearly distinguished from the H and D groups. This suggests that the metabolic profiles of the samples vary. Although the H and D groups seem to be closer, dandelion extracts may still alter the metabolic profile with a high-concentration diet, but the differences may not be as significant as those between the other groups. Conversely, Figure 6B shows a clear clustering for each diet with little overlap, while the L and D groups are separated but closer to one another than to the H group, which is separated. This suggests that rumen metabolites varied slightly between the groups.

The Venn diagram (Figure 6C) shows the common metabolites among the groups, with each shade of color denoting a separate comparison group. A total of 1097 metabolites were identified across the comparison groups; among them, 111 metabolites were shared by all comparison groups. There were 233 metabolites outside the overlapping areas for comparison groups L vs. H, 199 for comparison groups L vs. D, and 61 for comparison groups H vs. D. This suggests that the comparison groups L vs. H and L vs. D contain greater numbers of distinct differential metabolites. Figure 6D uses a volcano graphic to show the significant differences in metabolite levels between the H and D groups. Among them are metabolites with significant fold changes and statistical significance: 2946 unchanged (gray), 148 upregulated (red), and 205 downregulated (blue). One of the most significantly down-regulated metabolites found in the plot was 5-((6-((aminomethyl)amino)-1-oxohexyl)amino) pentanoic acid, whereas the most significantly upregulated metabolites were cyclohexa-2,4-dienylmethanol, 7-ethyl-5,6-dihydro-1,4-dimethylazulene, and trans-3-Hydroxy-L-proline. Whereas the downregulated metabolites might point to repressed metabolic activities, the upregulated metabolites might point to an upregulated pathway or higher metabolic activity in one scenario.

### 3.7. Differential Metabolite Enrichment Analysis in KEGG Pathways

**Figure 7:**
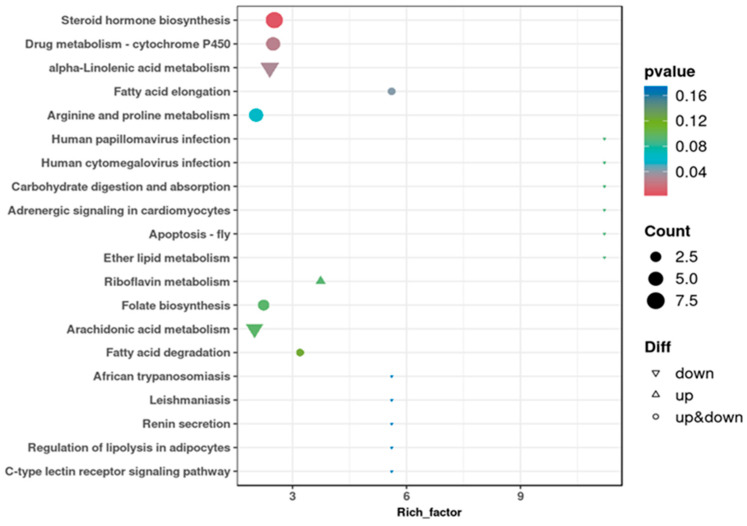
KEGG enrichment dot plot of differential metabolites. The graph’s points each represent KEGG pathways; the color depth of the points indicates the *p*-value; a lower *p*-value suggests a more accurate enrichment of differential metabolites in that pathway; a rich factor representing the proportion of all metabolites in that pathway that are identified as differential metabolites.

A KEGG enrichment dot map in Figure 7 indicates that the H and D groups have distinct levels of enrichment in some pathways linked to immunological response, metabolism, and cellular functions. Steroid hormone biosynthesis, drug metabolism-cytochrome P450, and alpha-linolenic acid metabolism are among the pathways that show high rich factors and substantial enrichment (*p* < 0.05), suggesting that these pathways may play prominent roles in differentiating between the two diet groups. The presence of both upregulated and downregulated pathways across metabolic processes, such as alpha-linolenic acid metabolism (down and *p* < 0.05), arachidonic acid metabolism (down), riboflavin metabolism (up), fatty acid elongation, arginine and proline metabolism, folate biosynthesis and fatty acid degradation, suggests a complex metabolic shift in response to dietary differences.

### 3.8. Correlation Analysis Between the Ruminal Fluid Parameters, Microbiome, and Metabolome in Dairy Goats

**Figure 8:**
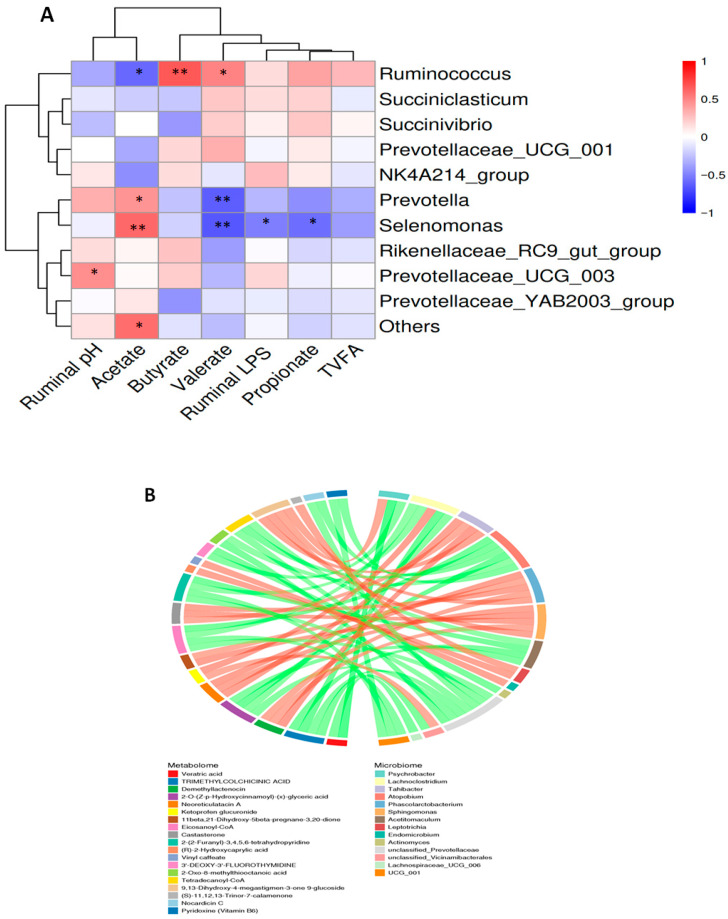
A correlation analysis between ruminal fluid parameters, differential metabolites, and differential microbial genera of dairy goats. (**A**) Correlation heatmap: The red squares signify positive association, whereas blue shows negative association. * Shows a significant (*p* < 0.05) difference between the groups; ** shows a highly significant (*p* < 0.01) difference between the groups. (**B**) Chord diagram: The chord diagram’s left and right halves represent the differential metabolite and the differential microorganism, respectively. Each chord indicates a significant correlation between the two, with a red chord representing a positive correlation and a green chord representing a negative correlation.

Figure 8A shows the correlation between ruminal microbial genera and ruminal parameters, including pH, LPS concentration, and fermentation products. Positive correlations were observed between *Ruminococcus*, Butyrate, and Valerate, while *Prevotellaceae_UCG_003* was positively correlated with ruminal pH (*p* < 0.05 and *p* < 0.01). Significant positive correlations were also found between *Selenomonas*, *Prevotella*, and *Others* genera and Acetate (*p* < 0.01 and *p* < 0.05). Conversely, negative correlations were observed between *Prevotella* and Valerate; between *Selenomonas* and Valerate, Propionate, and LPS concentrations; and between *Ruminococcus* and Acetate (*p* < 0.01 and *p* < 0.05). Figure 8B presents a correlation chord diagram between 14 differential microbial genera and 19 differential metabolites (H vs. D groups), showing positive correlations such as *Phascolarctobacterium* with 9,13-Dihydroxy-4-megastigmen-3-one9-glucoside, and *Sphingomonas* with (R)-2-Hydroxycaprylic acid, and a negative associations such as *Unclassified_Prevotellaceae*, *UCG_001*, and *Lachnoclostridium* with Eicosanoyl-CoA, and *Acetitomaculum* and *Unclassified_Prevotellaceae* with Tetradecanoyl-CoA.

## 4. Discussion

The findings of this study support our study objective and confirm that dandelion extract can improve rumen function and microbial activity in dairy goats fed high-concentrate diet. The observed shifts in microbial communities, metabolites and immune indicators are consistent with the expected effects of plant-derived bioactive compounds. However, the small sample size may have limited the detection of some differences, so these results should be considered preliminary, and further studies with larger sample sizes are needed to confirm and expand on these observations.

### 4.1. Influence of Various Feeds on Feed Intake, Milk Yield, and Milk Quality in Dairy Goats

For optimal digestion, ruminants need to consume a balanced diet of energy and fiber. Concentrate-rich diets increase feed intake and energy availability by supplying readily fermentable carbohydrates [1]. Concerning our results, ruminants’ preference to ingest concentrate-rich feeds correlates with higher intake of energy-dense diets, and the lower feed intake in the L group may be due to its lower energy content. Moreover, supplementation with dandelion extracts has been shown to boost rumen fermentation by improving the abundance of rumen bacteria responsible for fiber digestion [14], attributable to its flavonoids that offer gastrointestinal health benefits, which could be the reason for the D group’s high feed intake. Furthermore, the elevated feed intake noted in the H and D groups during the experiment may result from the limited duration of concentrate feeding.

Based on the results, milk production in the L group, with a higher forage-to-concentrate ratio of 60:40, gradually declined, while the H and D groups, with a 35:65 ratio, showed increased milk production. This trend is closely linked to the energy levels in the diet. A net energy content of 1.54 Mcal/kg results in higher yields than 1.40 Mcal/kg, according to Zhou et al. [16], and larger concentrate-to-forage ratios (e.g., 65:35) considerably boost milk yield as compared to lower ratios (e.g., 46:54) [17]. However, because of inflammation, cows fed high-concentrate diets for extended periods of time may eventually produce less milk [17]. Dandelion extracts had a modest effect on dairy goat milk yield, according to the study’s observation of slight variations in milk production between the H and D groups. There are not enough in vivo studies on dandelion and its extracts in this area but comparable research on dairy cows has indicated that dandelion extracts might have a minor impact on milk production [12].

The present study found that the H and D groups showed higher levels of lactose and protein, particularly towards the end of the study. This fits with what Zain et al. [18] found, namely, that higher-concentrate diets encourage microbial protein synthesis in the rumen, which leads to more protein in the milk. This is because they make more nutrients and fermentable carbohydrates available. Additionally, studies have linked increased energy intake to elevated lactose synthesis in dairy cows [19]. The D group had almost no effect on the overall quality of the milk. The findings of Zhang et al. [12] are comparable in that they discovered that dandelion extracts had minimal impacts on the composition of milk, mostly resulting in slight increases in the quantity of fat and protein in a high-concentrate diet.

### 4.2. Influence of Various Feeds on Ruminal pH, Ruminal LPS Concentrations, and Ruminal Fermentation Parameters in Dairy Goats

The reduction in rumen pH observed in the H group compared to the L group supports the established link between high-concentrate diets and lower rumen pH. The reason is mainly that rapidly fermentable carbohydrates in concentrates produce more volatile fatty acids [2]. A decrease in pH like this could be a sign of subacute ruminal acidosis, a disorder in which the rumen’s buffering ability is exceeded by excessive volatile fatty acid accumulation [3]. Although the D group showed a numerically higher pH than the H group, this difference was not statistically significant; therefore, no conclusions were drawn regarding the effect of dandelion extract on pH. Furthermore, LPS, which is the main component of the Gram-negative bacteria cell wall, can pass through the circulatory system and causes inflammation when the ruminal pH drops due to acidosis brought on by a high-concentrate diet [3]. The results of the current study show that the ruminal LPS concentration was marginally lowered when the dandelion extracts were added to the existing high-concentrate diet. The trend of the results aligned with the findings of Zhang et al. [12], but the level of significance differed, as it was found that adding dandelion extracts to a high-concentrate diet decreased the amount of ruminal LPS in dairy cows.

Ruminants can use a diet high in fiber to create VFAs, which are the primary sources of energy and contribute to production demand [20]. However, ruminants may suffer from acidosis due to the excessive generation of VFAs [2]. While the addition of dandelion extracts to a high-concentrate diet decreased the levels of the aforementioned fermentation parameters, the current investigation found that the L group had lower levels of valerate, propionate, and total VFAs. The in vitro culture analysis showed that the plant extract with a high content of flavonoids lowers the level of total VFAs obtained from culture ruminal microflora from dairy cows [21], and the addition of flavonoids in a high-concentrate diet may be effective in improving ruminal fermentation parameters and lowering the effect of ruminal acidosis [22]. Zhang et al. [23] showed that the levels of valerate, propionate, and total VFAs were decreased in dairy goats fed a low-concentrate diet. Furthermore, the amount of acetate was significantly higher in the L and D groups than in the H groups, which was comparable to Zhang et al. [23], who noticed an increase in acetate levels in dairy goats fed a low-concentrate diet. While dairy cows’ rumen fermentation also becomes more efficient, ruminal fermentation parameters like acetate are elevated when dandelion extracts are supplemented [14].

### 4.3. Influence of Various Feeds on Serum Immunoglobulin, Serum Pro-Inflammatory Cytokines, and the Expression of a Gene Linked to Inflammation in Dairy Goats

Supplementation of dandelion extracts in a high-concentrate diet significantly promotes the level of serum IgA. Dandelion extracts contain a lot of phytochemicals like flavonoids, which may help stimulate the immune system and reduce inflammation [11]. IgA plays an important role in epithelial immunity by serving as the initial line of protection against pathogens at the surfaces of mucosa such as the gastrointestinal tract [24]. Dairy goats’ mucosal immune response may be enhanced by dandelion extract supplementation, as seen by the greater level of IgA seen in this study. On the other hand, Akhtar et al. [25] observed that higher levels of IL-1β and IL-6 could adversely influence milk production, milk quality, and ruminant health. The present study found that the addition of dandelion extracts to the high-concentrate diet results in a numerical decrease in the relative mRNA expression level of IL-1β and IL-6, proposing that phytogenic feed supplements similar to dandelion extracts may influence immunological regulation. Furthermore, flavonoids have been demonstrated to suppress inflammatory gene expression, control cytokine production, and inhibit NF-κB activation in an in vitro investigation of bovine mammary epithelial cells [26].

### 4.4. Influence of Various Feeds on Ruminal Microbial Composition in Dairy Goat

The rumen bacterial community performs binary important roles in ruminants, which involve the fermentation of structural carbohydrates and the synthesis of essential nutrients which are crucial for ruminant performance and health. Because of their functions in the breakdown of fiber and the synthesis of short-chain fatty acids, the phyla *Firmicutes* and *Bacteroidota* have been identified as essential components of the rumen microbiota in previous studies [14], and the present study confirms their dominance across all dietary groups. The increased Firmicutes/Bacteroidota ratio in the H group aligns with previous reports of high-concentrate diets promoting bacteria that ferment non-structural carbohydrates. The reduction in fiber-degrading genera such as *Prevotella* further supports this shift. Dandelion extract supplementation moderated this change, maintaining a microbial profile more conducive to fiber digestion. The relative abundance of *Proteobacteria* was significantly increased in the dandelion group as compared with other groups. *Proteobacteria* include rumen bacteria involved in important metabolic functions, particularly the fermentation of carbohydrates [27]. The higher levels of *Proteobacteria* observed in the present study may suggest a response to the higher levels of rapidly fermentable carbohydrates presented in the high-concentrate diet containing dandelion extracts, which may have a unique effect on the rumen microbiome. This was supported by a preceding study in dairy goats that reported that plant feed additives can stimulate proliferation of beneficial microbial populations and decrease harmful populations [28].

The present study indicated that *Prevotella*, the dominant genus in the L group, exhibited excellent adaptability to high-fiber diets. This genus has a variety of metabolic pathways that aid in the breakdown of complex polysaccharides included in low-concentrate diets. Betancur-Murillo et al. [29] reported that in a low-concentration diet, *Prevotella* is crucial for the fermentation of structural carbohydrates into volatile fatty acids, which are necessary for ruminant energy. Furthermore, decreased *Prevotella* abundance in the H group remains consistent with recent research indicating that the decline of *Prevotella* populations negatively impacts fiber digestion and overall gut health [30]. Prevotella levels increased when dandelion extracts were supplemented, indicating that the bioactive compounds, such as flavonoids, in this extract had a beneficial effect on the rumen microbiota by encouraging the breakdown of fiber and the generation of VFA. Consistently, alfalfa flavonoids have been demonstrated to increase the number of cellulolytic bacteria in dairy cows [31].

### 4.5. Influence of Various Feeds on Alpha Diversity and Beta Diversity of the Microbial Community in Dairy Goats

The microbiological richness and evenness of a given sample are frequently evaluated using alpha diversity indices such as Shannon, ACE, Simpson, Chao1, and others. It has been shown that high-concentrate diets drastically change the rumen environment, mostly by reducing the diversity of microorganisms. This is probably because they include more easily fermentable carbohydrates and less fiber. These dietary changes promote the proliferation of bacterial communities specialized in fermenting simple carbohydrates while suppressing cellulose-degraded bacteria. Empirical evidence consistently supports this observation, showing that high-concentrate diet not only reduces microbial diversity but also lowers pH, ultimately leading to decreased abundance and diversity of ruminal bacteria [32]. Similar microbial shifts occurred in the H group of our study, thereby contributing to lower species richness relative to the L group. It is well known that the flavonoids in dandelion may influence microbial populations, promoting healthy bacteria while inhibiting harmful ones [33]. The trend observed in the present study may be explained by the dandelion’s contribution to a more varied microbial population in the D group relative to the H group. The observed trends provide information about the rumen microbial community in goats, even if the changes in alpha diversity indices between the diet groups were not considered highly significant. The D group has higher alpha diversity than the H group, which implies that the dietary composition of dandelion extracts might promote a wider variety of rumen microflora.

In microbiome studies the beta diversity is an important metric since it quantifies the degree of variation in microbial communities between groups and individual samples. This may help the experts to know the additional information regarding the nutritional modification and ecological variables that impact the composition of rumen microflora. Since the dots in the PCA and PCoA graphs closely cluster around a single region, it appears that there are minimal differences in the rumen microbial communities amongst the goat groups in this investigation. The result is consistent with previous work of Li et al. [34], where it was proved that though certain nutritional approaches may influence the diversity of microbes in some circumstances, the general composition of the local ecosystem can stay constant. The degree to which nutritional content influences the number of microbes also varies depending on the individual ingredients in food and how they interact [35]. The present study found that beta diversity assessment has shown relatively slight differences in the rumen microbial composition of dairy goats across different feed groups. This discovery highlights the innate stability of ruminant bacterial communities even in the context of alterations in nutrition which advances our knowledge of how feed influences these populations.

### 4.6. Influence of Various Feeds on Dairy Goat Rumen Fluid Metabolites

Metabolites facilitate a more efficient and intuitive comprehension of biological processes and their causes. Metabolites in rumen fluid are important markers of ruminant health, nutrient absorption, and total production [36]. Our results showed that the metabolites in the different dietary groups differed slightly. Guo et al. [37] focused on the impact of a high-concentrate diet on rumen fluid metabolites and discovered that such a diet may cause a substantial change in the microbial population and their metabolic generation. Furthermore, addition of dandelion extracts in a high-concentrate diet could potentially enhance the production of beneficial metabolites while alleviating the impact of a high-grain diet. A volcano plot of metabolites revealed the presence of 7-Ethyl-5,6-dihydro-1,4-dimethylazulene as an expressively upregulated metabolite, which was particularly interesting given the known anti-inflammatory properties of azulene derivatives. Azules are commonly present in plants and have shown a great deal of biological activity, including anti-inflammatory qualities [38]. Also, trans-3-Hydroxy-L-proline was the most significantly upregulated metabolite observed in the present study. Both humans and animals contain Hydroxyproline, an amino acid metabolite that significantly contributes to various biological processes, thereby enhancing animal health and performance [39]. Supplementing dandelion extracts may enhance the production of beneficial metabolites by microbial metabolism in the rumen, and the presence of these metabolites indicates complex interactions between microbial communities and diets. By increasing the synthesis of these metabolites, dandelion phytochemicals may help dairy goats exposed to the dangers of a high-grain diet maintain metabolic stability.

### 4.7. Differential Metabolite Enrichment Analysis in KEGG Pathways

The inclusion of dandelion extracts in a high-concentrate diet significantly influences lipid metabolism. Similarly to the present study, previous research has revealed multiple bioactive compounds in dandelion, with a specific focus on flavonoids that may modulate lipid metabolism. According to Orzuna-Orzuna et al. [40], flavonoids raise the concentration of milk fat in dairy cows, while phenolic compounds like tannins may alter ruminal lipid metabolism and fatty acid profile of milk by increasing the concentration of some potentially advantageous fatty acids [41]. Additionally high-concentrate feed supports the metabolism of amino acids to satisfy the requirements of enhanced milk production and metabolic activity [23]. The different enrichment patterns between the groups that received a high-concentration diet with and without dandelion extracts suggest that dandelion extracts may influence the metabolism of amino acids by supplying bioactive chemicals that engage with metabolic pathways.

### 4.8. Correlation Analysis Between the Ruminal Fluid Parameters, Microbiome, and Metabolome in Dairy Goats

The relative abundance of *Prevotella* was shown to be significantly higher in the group that consumed a high-concentrate diet with dandelion extracts, and a significant beneficial relationship was also established between *Prevotella* and Acetate. The fermentation of structural carbohydrates by the genus *Prevotella* is well known for producing volatile fatty acids like acetate, which are essential for energy generation and also help ruminants meet their nutritional needs [29]. In the ruminal metabolome and microbiome, Firmicutes species were found to have favorable relationships with fermentation-associated components [14]. Sphingomonas and (R)-2-Hydroxycaprylic acid were found to be positively correlated in this study. More specifically, Sphingomonas is a Proteobacteria, and (R)-2-Hydroxycaprylic acid comes from a natural substance known as medium-chain fatty acid [42]. It is possible that dandelion extracts had an impact on the rumen microorganisms by encouraging the activity and capacity of Sphingomonas to metabolize (R)-2-hydroxycaprylic acid. In contrast, Phascolarctobacterium and 9,13-Dihydroxy-4-megastigmen-3-one9-glucoside showed a positive correlation. Phascolarctobacterium is a member of the phylum Firmicutes, which is involved in the fermentation of structural carbohydrates into a short-chain fatty acid [14] while 9,13-Dihydroxy-4-megastigmen-3-one9-glucoside is a member of the group of fatty acids, and it also contains glucoside, which is often found in plants and can be metabolized by gut microbes [43]. Such beneficial relationship implied that dandelion extracts might encourage Phascolarctobacterium to interact or metabolize glucoside compounds, which could impact the rumen’s synthesis of short-chain fatty acids.

Our findings illustrate an interconnected pathway: high-concentrate diets alter the ruminal microbiota, reducing fiber-degraders like *Prevotella* and increasing propionate and total VFAs, thereby elevating LPS through ruminal acidification. LPS translocation triggers systemic inflammation as reflected in serum cytokines and mammary gene expression. Metabolomic analysis revealed upregulated metabolites such as trans-3-Hydroxy-L-proline and azulene derivatives, which are linked to anti-inflammatory and metabolic pathways. Dandelion extract mitigated these effects by modulating microbiota composition, enhancing acetate, and promoting beneficial metabolites, thereby supporting ruminal homeostasis and immune function.

## 5. Conclusions

This study demonstrates that supplementing dairy goats with dandelion extract in a high-concentrate diet positively influenced immune function, enhanced ruminal fermentation, and promoted beneficial microbial and metabolic profiles. While parameters such as rumen pH, milk composition, and some cytokines were unaffected, the extract’s positive effects on serum IgA, VFAs, and microbial composition suggest it may support rumen health, metabolism, and overall performance of dairy goats. However, the small sample size and evaluation of a single dose limit the generalizability of these findings, so future studies with large sample sizes and varied doses are needed to confirm these effects and clarify the mechanisms involved.

## Figures and Tables

**Table 1:** Diet composition and nutrient profile of the entire mixed ration from different diets.

Items	Treatment		
	L	H	D
**Diet composition (% DM)**			
Alfalfa hay	24	16	15.84
Corn silage	24	11	10.89
Ryegrass	12	8	7.92
Corn	28	29.12	28.82
Wheat bran	0	17.87	17.69
Calcium bicarbonate	2.13	1.13	1.12
Soybean meal	5.73	7.88	7.81
DDGS	2.14	7	6.93
Premix	1	1	0.99
Salt	1	1	0.99
Dandelion extract	0	0	1
Forage to concentrate ratio	60:40	35:65	35:65
**Nutrient profile**			
Crude ash (% of DM)	11.23	12.01	11.42
Crude protein (% of DM)	15.06	16.41	16.22
Crude fat (% of DM)	1.40	2.20	1.82
Neutral detergent fiber (% of DM)	39.32	37.61	37.73
Acid detergent fiber (% of DM)	22.55	18.52	18.56
Metabolizable Energy (MJ/Kg)	14.84	15.21	15.05
Calcium (% of DM)	1.04	1.02	1.02
Phosphorous (% of DM)	0.33	0.39	0.37

The abbreviation L represents the low-concentrate diet group (control group) with an F:C of 60:40; H represents the high-concentrate diet group with an F:C of 35:65; and D represents the dandelion group with a % dandelion extracts group based on the H group; DDGS represents the distiller dried grain with soluble.

**Table 2:** Concentrations of primary target flavonoid metabolites in dandelion extracts, as determined by LC-MS.

Flavonoid Target Metabolites	Concentrations (μg/g)
Isorhamnetin	2021.81
Luteolin	1711.59
Puerarin	1112.88
Cynaroside	508.10
Genkwanin	352.48
Quercimeritrin	255.89
Hyperoside	232.40
Chrysin	206.49
Daidzin	177.18
3′-Methoxypuerarin	163.30
Scutellarein	83.63
Scutellarin	81.63
Apigenin	70.63
Linarin	66.79
Daidzein	61.21
Phlorizin	58.43
Rutin	42.53
Glycitin	39.58
Genistin	33.43
Quercitrin	33.12

**Table 3:** Real-time PCR primer list for target genes (inflammatory genes).

Target Gene	Primer Sequence (5′–3′)	Product Length (bp)	Reference/Gene Bank Accession No.
NF-κB	F: CTCACCAATGGCCTCCTCTC	179	XM_018043384.1
	R: ACACCCTCCCAGAATCCGTA		
TLR4	F: TTCGCATCTGGATAAATCCAGC	207	NM_001285574.1
	R: CTGAGAACCGAGAGCTGGGAC		
TNF-α	F: CAAGTAACAAGCCGGTAGCC	153	XM_005696606.3
	R: AGATGAGGTAAAGCCCGTCA		
IL-1β	F: CATGTGTGCTGAAGGCTCTC	172	XM_013967700.2
	R: AGTGTCGGCGTATCACCTTT		
IL-6	F: CCAATCTGGGTTCAATCAGG	240	NM_001285640.1
	R: ACCCACTCGTTTGAGGACTG		
IL-8	F: ATGGAACAATGTACATGTGACAC	367	XM_005681749.3
	R: CTGAGAGTTATTGAGAGTGGGC		
IL-10	F: TTAAGGGTTACCTGGGTTGC	237	XM_005690416.3
	R: CCCTCTCTTGGAGCATATTGA		
GAPDH	F: CGGCACAGTCAAGGCAGAGAAC	115	XM_005680968.3
	R: CACGTACTCAGCACCAGCATCAC		

Nuclear Factor Kappa B is written as NF-κB, Toll-like Receptor 4 as TLR4, Tumor Necrosis Factor Alpha as TNF-α, Interleukin 1beta as IL-1β, Interleukin 6 as IL-6, Interleukin 8 as IL-8, Interleukin 10 as IL-10, and GAPDH as Glyceraldehyde-3-phosphate dehydrogenase.

**Table 4:** Influence of various feeds on milk quality in dairy goats.

Items	Treatment			SEM	*p*-Value
	L	H	D		
Milk fat (%) W1	2.58	2.22	2.44	0.285	0.706
Milk fat (%) W2	2.42	2.29	2.36	0.226	0.930
Milk fat (%) W3	2.31	2.07	2.35	0.142	0.391
Milk fat (%) W4	2.34	2.18	2.41	0.149	0.594
Milk fat (%) W5	2.73	2.04	2.36	0.205	0.115
Non-Fat Solid (%) W1	7.78	8.21	8.10	0.200	0.337
Non-Fat Solid (%) W2	7.83	7.94	7.92	0.106	0.767
Non-Fat Solid (%) W3	7.63	7.88	7.80	0.184	0.625
Non-Fat Solid (%) W4	7.60	7.88	7.84	0.163	0.442
Non-Fat Solid (%) W5	7.64	7.82	7.74	0.147	0.719
Lactose (%) W1	3.49	3.68	3.63	0.090	0.349
Lactose (%) W2	3.51	3.55	3.56	0.049	0.742
Lactose (%) W3	3.42	3.53	3.50	0.082	0.647
Lactose (%) W4	3.41	3.52	3.53	0.073	0.460
Lactose (%) W5	3.43	3.47	3.51	0.066	0.748
Protein (%) W1	3.68	3.85	3.84	0.087	0.356
Protein (%) W2	3.76	3.75	3.71	0.051	0.751
Protein (%) W3	3.61	3.68	3.73	0.074	0.574
Protein (%) W4	3.60	3.71	3.73	0.077	0.447
Protein (%) W5	3.60	3.71	3.73	0.069	0.703

The abbreviation L represents the low-concentrate diet group (control group) with an F:C of 60:40; H represents the high-concentrate diet group with an F:C of 35:65; D represents the dandelion group with a % dandelion extracts group based on the H group; W1–W5 represents week 1 to week 5.

**Table 5:** Influence of various feeds on ruminal fermentation parameters in dairy goats.

Items	Treatment			SEM	*p*-Value
	L	H	D		
Acetate (mmol/L)	72.35 ^a^	32.76 ^b^	58.87 ^a^	7.12	0.013
Propionate (mmol/L)	31.51 ^B^	115.54 ^A^	82.25 ^A^	11.63	0.001
Butyrate (mmol/L)	28.32	51.36	34.34	7.96	0.142
Valerate (mmol/L)	0.44 ^b^	3.97 ^a^	1.74 ^ab^	0.87	0.044
Total VFA (mmol/L)	131.85 ^b^	204.64 ^a^	188.28 ^a^	15.89	0.014

Various letters in a particular row of data show a significant difference (*p* < 0.05). In contrast, the presence or absence of the same letter in the superscript shows no significant difference (*p* > 0.05). Also, the capital letter represents a significant difference (*p* < 0.01). The total VFA represents the total amounts of acetate, propionate, butyrate, and valerate.

**Table 6:** Microbial composition of dairy goat feed from various diets at both the phylum and the genus levels.

Items	Treatment			SEM	*p*-Value
	L	H	D		
**Phyla (%)**					
*Bacteroidota*	49.483	45.422	44.561	4.861	0.761
*Firmicutes*	41.629	48.831	44.288	5.202	0.627
*Proteobacteria*	2.108 ^b^	1.890 ^b^	6.875 ^a^	1.259	0.032
*Spirochaetota*	1.016	1.398	1.793	0.848	0.829
*Actinobacteriota*	1.530	0.661	0.207	0.496	0.415
*Patescibacteria*	0.381	0.771	0.736	0.226	0.474
*Synergistota*	1.371	0.004	0.105	3.080	0.381
*Cyanobacteria*	0.795	0.267	0.341	0.347	0.657
*Desulfobacterota*	0.546	0.304	0.311	0.120	0.423
*Fibrobacterota*	0.185	0.140	0.297	0.093	0.495
**Genera (%)**					
*Prevotella*	22.077 ^a^	8.508 ^b^	21.535 ^a^	2.658	0.010
*uncultured_rumen_bacterium*	6.180	12.487	7.080	2.141	0.127
*unclassified_Prevotellaceae*	7.709	11.682	5.013	2.192	0.181
*Prevotellaceae_UCG_001*	2.766	5.693	4.040	1.291	0.328
*Rikenellaceae_RC9_gut_group*	3.394	2.293	3.184	1.011	0.481
*Prevotellaceae_UCG_003*	1.604	1.588	0.870	0.606	0.677
*Prevotellaceae_YAB2003_group*	2.715	0.782	0.179	1.009	0.450
*unclassified_Muribaculaceae*	0.535	1.185	1.655	0.697	0.580
*Succiniclasticum*	2.646	7.355	6.319	1.874	0.304
*unclassified_Clostridia_UCG_014*	1.838	6.396	4.770	2.857	0.629
*Ruminococcus*	1.959 ^b^	6.927 ^a^	3.500 ^ab^	1.352	0.085
*Selenomonas*	4.443	0.838	1.760	1.111	0.171
*unclassified_Selenomonadaceae*	2.670	2.633	1.397	1.316	0.756
*NK4A214_group*	1.823	2.247	2.122	0.623	0.886
*unclassified_Lachnospiraceae*	4.255	0.736	0.611	1.073	0.233
*Agathobacter*	0.513	1.876	1.981	0.875	0.492
*Lachnospira*	1.681	0.865	1.528	0.719	0.760
*Christensenellaceae_R_7_group*	1.127	1.996	0.747	0.510	0.350
*Succinivibrio*	0.365	0.932	3.399	0.903	0.176
*Treponema*	1.000	1.387	1.773	0.848	0.830

Various letters in a particular row of data show a significant difference (*p* < 0.05). In contrast, the presence or absence of the same letter in the superscript shows no significant difference (*p* > 0.05).

## Data Availability

The original contributions presented in this study are included in the article. Further inquiries can be directed to the corresponding author.

## References

[1] Șonea C., Gheorghe-Irimia R.A., Dulaimi M.K.H.A., Udrea L., Tăpăloagă D., Tăpăloagă P.-R. (2024). Optimizing Feed Formulation Strategies for Attaining Optimal Nutritional Balance in High-Performing Dairy Goats in Intensive Farming Production Systems. Ann. Valahia Univ. Târgovişte Agric..

[2] Giger-Reverdin S. (2018). Recent Advances in the Understanding of Subacute Ruminal Acidosis (SARA) in Goats, with Focus on the Link to Feeding Behaviour. Small Rumin. Res..

[3] Monteiro H.F., Faciola A.P. (2020). Ruminal Acidosis, Bacterial Changes, and Lipopolysaccharides. J. Anim. Sci..

[4] Jin D., Chang G., Zhang K., Guo J., Xu T., Shen X. (2016). Rumen-Derived Lipopolysaccharide Enhances the Expression of Lingual Antimicrobial Peptide in Mammary Glands of Dairy Cows Fed a High-Concentrate Diet. BMC Vet. Res..

[5] Cochet F., Peri F. (2017). The Role of Carbohydrates in the Lipopolysaccharide (LPS)/Toll-Like Receptor 4 (TLR4) Signalling. Int. J. Mol. Sci..

[6] Nuñez A.J.C., Caetano M., Berndt A., Demarchi J.J.A.D.A., Leme P.R., Lanna D.P.D. (2013). Combined Use of Ionophore and Virginiamycin for Finishing Nellore Steers Fed High Concentrate Diets. Sci. Agric..

[7] Shao Y., Wang Y., Yuan Y., Xie Y. (2021). A Systematic Review on Antibiotics Misuse in Livestock and Aquaculture and Regulation Implications in China. Sci. Total Environ..

[8] Michalak M., Wojnarowski K., Cholewińska P., Szeligowska N., Bawej M., Pacoń J. (2021). Selected Alternative Feed Additives Used to Manipulate the Rumen Microbiome. Animals.

[9] Chagas M.D.S.S., Behrens M.D., Moragas-Tellis C.J., Penedo G.X.M., Silva A.R., Gonçalves-de-Albuquerque C.F. (2022). Flavonols and Flavones as Potential Anti-Inflammatory, Antioxidant, and Antibacterial Compounds. Oxid. Med. Cell. Longev..

[10] Paniagua M., Crespo J., Arís A., Devant M. (2019). Citrus Aurantium Flavonoid Extract Improves Concentrate Efficiency, Animal Behavior, and Reduces Rumen Inflammation of Holstein Bulls Fed High-Concentrate Diets. Anim. Feed Sci. Technol..

[11] Yan Q., Xing Q., Liu Z., Zou Y., Liu X., Xia H. (2024). The Phytochemical and Pharmacological Profile of Dandelion. Biomed. Pharmacother..

[12] Zhang Y., Mgeni M., Xiu Z., Chen Y., Chen J., Sun Y. (2024). Effects of Dandelion Extract on Promoting Production Performance and Reducing Mammary Oxidative Stress in Dairy Cows Fed High-Concentrate Diet. Int. J. Mol. Sci..

[13] Kim S., Jo Y., Jeong S., Kim Y., Han J. (2024). An Investigation of Antioxidative and Anti-Inflammatory Effects of Taraxacum Coreanum (White Dandelion) in Lactating Holstein Dairy Cows. J. Adv. Vet. Anim. Res..

[14] Li Y., Lv M., Wang J., Tian Z., Yu B., Wang B., Liu J., Liu H. (2020). Dandelion (Taraxacum Mongolicum Hand.-Mazz.) Supplementation-Enhanced Rumen Fermentation through the Interaction between Ruminal Microbiome and Metabolome. Microorganisms.

[15] National Research Council (2007). Nutrient Requirements of Small Ruminants: Sheep, Goats, Cervids, and New World Camelids.

[16] Zhou J., Dong G., Ao C., Zhang S., Qiu M., Wang X., Wu Y., Erdene K., Jin L., Lei C. (2014). Feeding a High-Concentrate Corn Straw Diet Increased the Release of Endotoxin in the Rumen and pro-Inflammatory Cytokines in the Mammary Gland of Dairy Cows. BMC Vet. Res..

[17] Abaker J.A., Xu T.L., Jin D., Chang G.J., Zhang K., Shen X.Z. (2017). Lipopolysaccharide Derived from the Digestive Tract Provokes Oxidative Stress in the Liver of Dairy Cows Fed a High-Grain Diet. J. Dairy Sci..

[18] Zain M., Tanuwiria U.H., Syamsu J.A., Yunilas Y., Pazla R., Putri E.M., Makmur M., Amanah U., Shafura P.O., Bagaskara B. (2024). Nutrient Digestibility, Characteristics of Rumen Fermentation, and Microbial Protein Synthesis from Pesisir Cattle Diet Containing Non-Fiber Carbohydrate to Rumen Degradable Protein Ratio and Sulfur Supplement. Vet. World.

[19] Sammad A., Wang Y.J., Umer S., Lirong H., Khan I., Khan A., Ahmad B., Wang Y. (2020). Nutritional Physiology and Biochemistry of Dairy Cattle under the Influence of Heat Stress: Consequences and Opportunities. Animals.

[20] Owens F.N., Basalan M., Millen D.D., De Beni Arrigoni M., Lauritano Pacheco R.D. (2016). Ruminal Fermentation. Rumenology.

[21] Kim E.T., Guan L.L., Lee S.J., Lee S.M., Lee S.S., Lee I.D., Lee S.K., Lee S.S. (2015). Effects of Flavonoid-Rich Plant Extracts on In Vitro Ruminal Methanogenesis, Microbial Populations and Fermentation Characteristics. Asian-Australas. J. Anim. Sci..

[22] Onu E., Ugwoke J., Edeh H., Onu M., Onyimonyi A. (2024). A Review: Flavonoid; A Phyto-Nutrient and Its Impact in Livestock Animal Nutrition. World J. Adv. Res. Rev..

[23] Zhang J., Shi H.T., Wang Y.C., Li S.L., Cao Z.J., Yang H.J., Wang Y.J. (2020). Carbohydrate and Amino Acid Metabolism and Oxidative Status in Holstein Heifers Precision-Fed Diets with Different Forage to Concentrate Ratios. Animal.

[24] Li Y., Jin L., Chen T. (2020). The Effects of Secretory IgA in the Mucosal Immune System. BioMed. Res. Int..

[25] Akhtar M., Guo S., Guo Y., Zahoor A., Shaukat A., Chen Y., Umar T., Deng P.G., Guo M. (2020). Upregulated-Gene Expression of pro-Inflammatory Cytokines (TNF-α, IL-1β and IL-6) via TLRs Following NF-κB and MAPKs in Bovine Mastitis. Acta Trop..

[26] Jiang M., Lv Z., Huang Y., Cheng Z., Meng Z., Yang T., Yan Q., Lin M., Zhan K., Zhao G. (2022). Quercetin Alleviates Lipopolysaccharide-Induced Inflammatory Response in Bovine Mammary Epithelial Cells by Suppressing TLR4/NF-κB Signaling Pathway. Front. Vet. Sci..

[27] Zhang Y.K., Zhang X.X., Li F.D., Li C., Li G.Z., Zhang D.Y., Song Q.Z., Li X.L., Zhao Y., Wang W.M. (2021). Characterization of the Rumen Microbiota and Its Relationship with Residual Feed Intake in Sheep. Animal.

[28] Foksowicz-Flaczyk J., Wójtowski J.A., Danków R., Mikołajczak P., Pikul J., Gryszczyńska A., Łowicki Z., Zajączek K., Stanisławski D. (2022). The Effect of Herbal Feed Additives in the Diet of Dairy Goats on Intestinal Lactic Acid Bacteria (LAB) Count. Animals.

[29] Betancur-Murillo C.L., Aguilar-Marín S.B., Jovel J. (2022). Prevotella: A Key Player in Ruminal Metabolism. Microorganisms.

[30] Chen X., Su X., Li J., Yang Y., Wang P., Yan F., Yao J., Wu S. (2021). Real-Time Monitoring of Ruminal Microbiota Reveals Their Roles in Dairy Goats during Subacute Ruminal Acidosis. npj Biofilms Microbiomes.

[31] Zhan J., Liu M., Su X., Zhan K., Zhang C., Zhao G. (2017). Effects of Alfalfa Flavonoids on the Production Performance, Immune System, and Ruminal Fermentation of Dairy Cows. Asian-Australas. J. Anim. Sci..

[32] Grilli D.J., Fliegerová K., Kopečný J., Lama S.P., Egea V., Sohaefer N., Pereyra C., Ruiz M.S., Sosa M.A., Arenas G.N. (2016). Analysis of the Rumen Bacterial Diversity of Goats during Shift from Forage to Concentrate Diet. Anaerobe.

[33] Kibegwa F.M., Bett R.C., Gachuiri C.K., Machuka E., Stomeo F., Mujibi F.D. (2023). Diversity and Functional Analysis of Rumen and Fecal Microbial Communities Associated with Dietary Changes in Crossbreed Dairy Cattle. PLoS ONE.

[34] Li H., Yang E., Zhang S., Zhang J., Yuan L., Liu R., Ullah S., Wang Q., Mushtaq N., Shi Y. (2020). Molecular Characterization of Gut Microbial Shift in SD Rats after Death for 30 Days. Arch. Microbiol..

[35] Henderson G., Cox F., Ganesh S., Jonker A., Young W., Janssen P.H. (2015). Rumen Microbial Community Composition Varies with Diet and Host, but a Core Microbiome Is Found across a Wide Geographical Range. Sci. Rep..

[36] Zhang H., Tong J., Zhang Y., Xiong B., Jiang L. (2020). Metabolomics Reveals Potential Biomarkers in the Rumen Fluid of Dairy Cows with Different Levels of Milk Production. Asian-Australas. J. Anim. Sci..

[37] Guo C., Sun D., Wang X., Mao S. (2019). A Combined Metabolomic and Proteomic Study Revealed the Difference in Metabolite and Protein Expression Profiles in Ruminal Tissue From Goats Fed Hay or High-Grain Diets. Front. Physiol..

[38] Akram W., Tagde P., Ahmed S., Arora S., Emran T.B., Babalghith A.O., Sweilam S.H., Simal-Gandara J. (2023). Guaiazulene and Related Compounds: A Review of Current Perspective on Biomedical Applications. Life Sci..

[39] Hu S., He W., Wu G. (2022). Hydroxyproline in Animal Metabolism, Nutrition, and Cell Signaling. Amino Acids.

[40] Orzuna-Orzuna J.F., Dorantes-Iturbide G., Lara-Bueno A., Chay-Canul A.J., Miranda-Romero L.A., Mendoza-Martínez G.D. (2023). Meta-Analysis of Flavonoids Use into Beef and Dairy Cattle Diet: Performance, Antioxidant Status, Ruminal Fermentation, Meat Quality, and Milk Composition. Front. Vet. Sci..

[41] Frutos P., Hervás G., Natalello A., Luciano G., Fondevila M., Priolo A., Toral P.G. (2020). Ability of Tannins to Modulate Ruminal Lipid Metabolism and Milk and Meat Fatty Acid Profiles. Anim. Feed Sci. Technol..

[42] Wang B., Sun Z., Tu Y., Si B., Liu Y., Yang L., Luo H., Yu Z. (2021). Untargeted Metabolomic Investigate Milk and Ruminal Fluid of Holstein Cows Supplemented with Perilla Frutescens Leaf. Food Res. Int..

[43] Biernat K.A., Li B., Redinbo M.R. (2018). Microbial Unmasking of Plant Glycosides. mBio.

